# The Effect of Exercise Program Interventions on Frailty, Clinical Outcomes, and Biomarkers in Older Adults: A Systematic Review

**DOI:** 10.3390/jcm13216570

**Published:** 2024-11-01

**Authors:** Adchara Prommaban, Sasiwimon Moonkayaow, Phichayut Phinyo, Penprapa Siviroj, Wachiranun Sirikul, Peerasak Lerttrakarnnon

**Affiliations:** 1 Aging and Aging Palliative Care Research Cluster, Faculty of Medicine, Chiang Mai University, Chiang Mai 50200, Thailand; adchara.p@cmu.ac.th (A.P.); penprapa.s@cmu.ac.th (P.S.); wachiranun.sir@cmu.ac.th (W.S.); 2 Department of Family Medicine, Faculty of Medicine, Chiang Mai University, Chiang Mai 50200, Thailand; 3 Sanpatong Hospital, Sanpatong, Chiang Mai 50120, Thailand; sasiwi_mon@hotmail.com; 4 Center for Clinical Epidemiology and Clinical Statistics, Faculty of Medicine, Chiang Mai University, Chiang Mai 50200, Thailand; phichayut.phinyo@cmu.ac.th; 5 Department of Biomedical Informatics and Clinical Epidemiology, Faculty of Medicine, Chiang Mai University, Chiang Mai 50200, Thailand; 6 Department of Community Medicine, Faculty of Medicine, Chiang Mai University, Chiang Mai 50200, Thailand

**Keywords:** frailty, older adults, exercise program, all settings, biomarkers

## Abstract

**Background:** Frailty is characterized by the decreased ability in older adults to handle daily or acute stressors due to age-related declines in physiological reserve and organ system performance. This condition results from the interaction of multiple physiological pathways and changes in biomarkers. Exercise programs are currently recommended to promote “healthy aging” in frail older adults. **Objective:** This systematic review aimed to evaluate the effectiveness of exercise program interventions in improving outcomes related to frailty, physical function, cognitive performance, and biomarkers in frail older adults. **Methods:** This study was designed according to the PRISMA guidelines. A systematic search was conducted in PubMed, Embase, and Scopus for studies published between 2014 and 2024. Two independent reviewers extracted data, with disagreements resolved by a third reviewer. Randomized controlled trials involving pre-frail or frail older adults aged 60 and above in all settings were included. The focus was on the impact of exercise programs, especially multicomponent interventions, on frailty outcomes and biomarkers. **Results:** Nine studies involving a total of 2083 participants met the inclusion criteria. The age range of participants was 65.35 ± 5.15 to 86.7 ± 4.00 years, with 64.7% being female. The results demonstrated that multicomponent exercise programs significantly improved frailty status, enhanced physical and cognitive function, and improved emotional well-being. Additionally, these programs led to significant reductions in inflammatory biomarkers, such as IL-6 and TNF-α, which are associated with frailty. **Conclusion:** This review highlights the beneficial effects of multicomponent exercise programs on pre-frail and frail older adults, providing evidence that these interventions improve physical and cognitive functions and emotional well-being, and reduce inflammation. These findings offer valuable insights into developing targeted interventions to manage frailty in clinical practice.

## 1. Introduction

The World Health Organization (WHO) generally classifies adulthood from various references and guidelines, defining older adults as those 60 years and older [1,2,3]. The WHO predicts that the global population aged 60 and above will increase from 12% in 2015 to 22% by the year 2050, leading to a “super-aged society” in which the aged population is more than 21% [3,4]. Aging is a natural and complex biological process that has significant implications for the strategic development and implementation of healthcare and social welfare services [5]. Frailty, a condition characterized by reduced physiological reserves and a greater vulnerability to environmental stresses, is one of the most significant problems associated with aging [6,7]. Frailty is defined by two principal models: the phenotypic model, described by Fried and colleagues, which focuses on physical traits such as muscle weakness, exhaustion, and a decrease in physical activity; and the cumulative deficit model, which considers the accumulation of health deficits, including chronic diseases and impairments [6,8]. Both models are widely used in the assessment of frailty, offering complementary perspectives on this complex syndrome. Frailty is more than solely aging; it is a multidimensional syndrome influenced by genetic, environmental, and behavioral variables, as well as biological and functional variables. The presence of frailty in older adults not only has a negative impact on their overall quality of life, but also places a significant strain on healthcare resources as it is associated with heightened susceptibility to falls, hospitalizations, and disability [9,10]. The prevalence of frailty among older adults ranges between 5 and 10%, with higher rates observed in hospital settings and long-term healthcare organizations [11,12]. It is more common in countries with lower to intermediate incomes and among immigrant populations [13].

Biomarkers are characteristics of physiological dysregulation that could help in the diagnosis, monitoring, clinical decision-making, and assessment of the intervention efficacy of individual medical conditions. Some biomarkers provide valuable information about physiological processes related to frailty [14]. Studies have identified biological processes related to frailty such as musculoskeletal changes; alterations in endocrine, immune, metabolic markers, serum markers, and hormones; and chronic inflammation [14,15,16,17,18].

The WHO defines healthy aging as the ongoing progression and preservation of the physical and cognitive capabilities necessary for overall well-being in older individuals [2]. Functional ability refers to possessing the necessary capacities that allow individuals to engage in activities and achieve what they consider valuable [2]. In 2019, the WHO issued the Integrated Care of Elderly People (ICOPE) approach, which includes the assessment of intrinsic capacity [19]. One of the crucial elements of intrinsic capacity, specifically mobility or locomotor capacity, is vital for older adults in preserving their well-being and preventing dependency on assistance [20]. Certainly, the most effective strategy for preventing frailty and reducing the risk of falls is by undergoing managed and personalized exercise [21]. In 2020, the WHO recommended moderate to vigorous intense physical activity ≥ 3 times a week, focusing on functional balance and strength training to prevent falls in older adults and those with chronic diseases [22].

Physical activity includes any activity that requires energy consumption and muscle contraction, such as walking, gardening, and housework, with varying intensity, and often occurs spontaneously without being part of a regular routine. However, exercise is a subset of physical activity that involves planned, scheduled, repetitive, and purposeful physical activity to improve or maintain physical performance, such as jogging, cycling, and aerobic activities [23]. Many findings have demonstrated that multicomponent exercise and combined exercise reduce frailty in older adults. A multicomponent exercise program has a variety of physical aspects that integrate strength, aerobic, balance, and flexibility training and enhance muscle strength, endurance, stability, and mobility in older adults, whereas combined exercise programs usually include various components of exercise but may emphasize one component more significantly, such as combining strength training and aerobic exercise or adding flexibility to aerobic workouts [24]. Physical activity and exercise have been shown to have significant effects on various physiology and biochemistry in older adults, including chronic inflammation, mitochondrial dysfunction, the release of myokines, autophagy, oxidative damage, and insulin-like growth factor signaling [25,26]. Exercise interventions in the research have explored changes in blood biomarkers of physiological responses. A Korean study found that a combination of anaerobic and aerobic exercise improves insulin resistance and promotes the secretion of hormones associated with aging, such as the growth hormone (GH), insulin-like growth factor 1 (IGF-1), and dehydroepiandrosterone sulfate (DHEA-S), in older women [27]. Moreover, a previous clinical trial study described that a multicomponent exercise program delays frailty by improving physical performance and reducing inflammatory biomarkers such as interleukin-6 (IL-6) and the C-reactive protein (CRP) in frail older adults [28]. Furthermore, multicomponent exercise programs can improve markers of oxidative stress and antioxidant capacity, which can help mitigate the negative effects of frailty on health and well-being [26].

This systematic review investigated the effectiveness of exercise programs in improving various outcomes in older adults, including frailty, physical function, cognitive function, and biomarkers. The available evidence on exercise interventions for frail older adults did not provide clear guidance on the most effective program variable. The most optimal exercise program intervention, as well as the outcomes and biomarkers associated with frailty, remain uncertain. This study aims to investigate the potential benefits of an exercise program and evaluate frailty status, physical performance, and cognitive function as main outcomes, with biomarkers as surrogate outcomes.

## 2. Materials and Methods

This systematic review followed the Preferred Reporting Items for Systematic Reviews and Meta-analysis (PRISMA) guidelines of 2020 [29]. This study is registered on the International Prospective Register of Systematic Reviews (PROSPERO) ID: CRD42023422760.

### 2.1. Eligibility Criteria

The inclusion criteria for the studies were as follows: (1) older adults who are 60 years or older; (2) older adults identified as frail or pre-frail based on a validated scale and/or questionnaires; (3) availability for intervention with more than two types of physical activity or an exercise program including multicomponent exercise. These studies were compared studies on with older adults receiving standard care, undertaking usual exercise, or who do not undergo any intervention or therapy components. The variable outcomes included frailty status, physical performance, cognitive functions, functional capacity, depression, and others. The surrogate outcomes were identified biomarkers associated with frailty. The inclusion criteria also included randomized controlled trials studies, all-setting studies, and studies published in the English language. Non-randomized controlled trials and non-human studies were excluded.

### 2.2. Search Strategy

We carried out a systematic search from 3 databases, including PubMed, Embase, and Scopus. Data were selected from all the research outputs from January 2014 to June 2024. All searches were conducted using specific search terms or keywords of the title/abstract that related to the research topic followed by (“frailty” OR “frail”) AND (“elderly” OR “older” OR “older adults” OR “older person”) AND (“physical activity” OR “exercise program” OR “multicomponent exercise” OR “multimodal exercise” OR “combined exercise” OR “training”) AND (“biomarkers” OR “blood biomarkers” OR “serum” OR “hormonal biomarkers” OR “metabolic biomarkers”).

### 2.3. Selection Process

All data searches from three 3 databases were compiled in the Endnote software version 21 to manually remove any duplicate entries during the screening process. The remaining results were uploaded to the Rayyan web-based software, a tool specifically designed for systematic reviews [30]. Two independent reviewers (AP and SM) conducted the screening process, examining the titles and abstracts of the articles obtained from the searches. A third reviewer (WS) was consulted to reach a consensus and resolve any conflicts in cases where there were discrepancies or disagreements between the reviewers. Throughout the screening process, the reasons for excluding titles and abstracts were recorded for transparency and documentation purposes. The eligible studies were selected for full-text assessment. The full reports of titles and abstracts that met the inclusion criteria were reviewed independently by two reviewers (AP and SM). Any discrepancies were resolved by a third reviewer (WS). The reasons for excluding full-text articles were recorded. To document the study selection process and provide a comprehensive overview of the included and excluded studies, a PRISMA-compliant flowchart was created to summarize the step-by-step process of study selection and was included in the final report of this study.

### 2.4. Data Collection Process and Data Items

A standardized data extraction form was filled out. Two independent reviewers (AP and SM) independently extracted the predetermined data. In the event of any disagreements, a third reviewer (WS) resolved any problems. The following data of each study were extracted: author, country, year of publication, patients characteristics (age, sex, and other details), the number of patients included, the type and duration of the exercise program intervention, frailty measurement tools, and outcomes/significant findings (biomarkers and frailty outcomes). In cases where there were conflicts or discrepancies regarding specific studies, these issues were resolved through discussion and consultation among the reviewers involved in the data extraction process. A third reviewer was consulted to ensure a consensus was reached and any discrepancies were appropriately addressed.

## 3. Results

### 3.1. Study Characteristics

**Figure 1:**
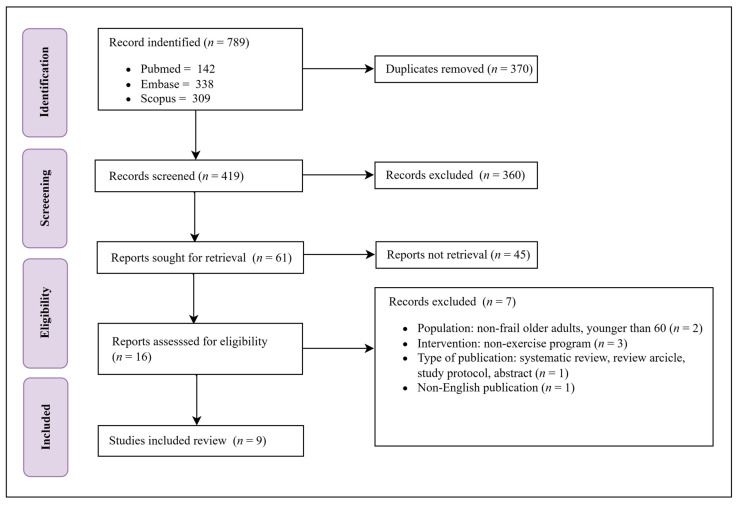
A flow diagram of the literature search and data selection.

A total of 789 studies were initially collected throughout the search (142, 338, and 309 studies from PubMed, Embase, and Scopus, respectively). Out of them, 370 studies were identified as duplicates and were therefore removed. The topic and abstract of the remaining 419 studies were evaluated. Following the exclusion of 360 records, 61 reports were sought for retrieval, of which 45 were not retrievable. For eligibility assessment, 16 studies were reviewed against the eligibility criteria and then 7 studies were excluded because they were either not entire texts or did not meet the eligibility criteria due to incompatible factors. As a result, there were nine studies that met the criteria for the systematic review [28,31,32,33,34,35,36,37,38] (Figure 1).

### 3.2. Participants

The included studies involved a total of 2083 older adults with pre-frailty and frailty, with 64.7% being female. The mean age ranged from 67.68 ± 5.19 to 86.7 ± 4.0 years in the intervention groups, and from 65.35 ± 5.15 to 83.3 ± 8.2 years in the control groups. The eligible studies were conducted in the following countries; Spain [31], the USA [32], Thailand [28], Portugal [33,34,35,36], Singapore [37], and China [38]. Almost all studies included participants who were both old men and women [28,31,32,35,36,37,38], and only two studies from Portugal [33,34] focused on solely women who were identified as pre-frailty and frailty. The study from the USA was found to be a multicenter study from eight US field centers [32]. The findings provided by this result included a total of six studies on physical frailty and three studies on cognitive frailty (Table 1).

### 3.3. Frailty Criteria and Assessment Tools

The frailty status in this study was identified as pre-frail or frail using a validated scale and/or questionnaires. Fried’s frailty phenotype (FFP) [28,31,33,34,35,36] was the main assessment tool for frailty identification. The Edmonton frailty scale (EFS) [31,38], the study of osteoporotic fractures (SOF) index [32], and the frail scale [37] were also used for fraility measurement.

Other assessments, including physical performance, cognition, psychometric scale, social, nutrition, and quality of life tests, were determined against the eligibility criteria and outcomes measurements. Many tools for physical performance tests were used to measure physical fitness in older adults, including the short physical performance battery (SPPB) [31,32,37], timed-up-and-go (TUG) [28,31], the hand grip strength test (HGT) [28,34,37], and others [28,31,33,34,35,36,37], which are shown in Table 1. For cognitive assessment, mini-mental state examination (MMSE) [31,33,34,35,36], the modified mini-mental state examination (3MSE) [32], the mini-mental state examination—Thai version (MMST10) [28], and the MoCA [37,38] were used. The nutrition status was determined by the mini nutritional assessment (MNA) [33,34,35,36,37]. Other assessments were used for social and quality of life tests [28,31,34], as presented in Table 1.

### 3.4. Exercise Program Intervention

The characteristics of the exercise program interventions are detailed in Table 2. In summary, most interventions were multicomponent exercise programs that included aerobic, resistance, balance, and flexibility training [28,31,32,36,37,38]. One study focused on chair-based exercises, emphasizing flexibility, strength, and balance [34]. Another study implemented a three-phase exercise program consisting of elastic-band exercises, exercise detraining, and multicomponent exercises [35]. Additionally, one study introduced a chair multimodal exercise program, which involved two sessions of functional circuit training [33]. The duration of these exercise programs ranged from approximately 14 to 40 weeks [28,31,33,34,35,36,38]. Two studies employed prolonged data collection durations: one lasted 12 months [37] while the other, which was a multicenter investigation, covered a period of 24 months [32].

### 3.5. Outcomes

#### 3.5.1. Primary Outcome (Clinical Outcomes)

The multicomponent exercise programs had significant benefits for older people, enhancing their overall health and well-being (Table 1). The interventions, comprising a combination of aerobic exercises, weight training, balancing exercises, and flexibility routines, proved very beneficial in improving physical, emotional, and social well-being. The interventions resulted in significant improvements in functional performance tests such as the SPPB and TUG tests, indicating a reduction in frailty severity [31]. In addition, the interventions proved to be effective in reducing frailty. Other studies also reported a primary outcome of reducing frailty through exercise interventions, demonstrated by improvements in HGS, walking speed, and overall physical activity [28,35]. A number of studies indicate that functional activity assessments, such as HGS, the gait speed test GST, and the BBS, demonstrate significant improvements after exercise interventions in pre-frail and frail older adults [33,34]. Furthermore, the exercise program interventions showed significant improvements in cognitive function, which was assessed through the MMSE, and the MoCA [31,33,38]. Another study found that multicomponent exercise programs significantly reduced cognitive frailty, as evidenced by 3MSE scores [32].

Along with improving physical health and cognition, the multicomponent exercise interventions also had positive impacts on emotional and social well-being, measured by the SWLS [34]. Participants noticed significant improvements in mood and a decrease in depression symptoms, as shown through the the GDS [28]. One study reported potential enhancements in the emotional state of participants, measured through scales such as the GDS and the Duke Social Support Index [31]. It is indicated that the exercise group experienced reduced symptoms of depression and improved social interactions, contributing to better overall emotional health.

#### 3.5.2. Secondary Outcome (Biomarker Associated Frailty)

Biomarker-associated frailty can be summarized and categorized, as seen in Table 3. There are several categories of biomarkers, including inflammatory, oxidative stress, coagulation, neurotrophic factor, hormonal, immune function, metabolic and protein, and hematological biomarkers. Biomarkers are determined in serum, plasma, and saliva. A variety of biomarkers significantly contribute to the development and progression of frailty in older adults.

Several studies have focused on inflammatory biomarkers, particularly IL-6, which demonstrates both pro-inflammatory and anti-inflammatory activities. The research indicates that IL-6 is associated with age-related diseases and frailty, with levels mostly decreased through exercise program interventions [28,32,35], as shown in Table 1 and Table 3. In addition, study results show that IL-10, an anti-inflammatory cytokine, is involved in modulating the inflammatory response linked to frailty [35]. One study revealed that exercise programs reduce CRP levels, highlighting their significance in the management of frailty [28]. Likewise, one study also demonstrated that TNF-α levelssignificantly decreased [37]. Other inflammatory biomarkers such as IL-1B, IL-2, IL-4, IL,8, IFN-γ, MPO, and GDF-15 were also determined but did not show a significant decrease. Protein carbonylation and MDA, biomarkers of oxidative stress, contributed to frailty [31,38]. According to one study, the exercise program had the potential to significantly increase SOD levels as well, as decrease MDA and 8-iso-PGF2α levels [38]. The BDNF supported neuronal growth and cognitive function, indicating reversed frailty and improved cognition [31]. D-dimer, a coagulation biomarker, was reduced with exercise interventions, suggesting improved frailty outcomes [31]. The hematological markers indicated that exercise showed a pro-immune effect and sustained the levels of MCH and MCHC, consequently promoting healthy aging [36].

For salivary biomarkers, including hormonal biomarkers including testosterone and cortisol it was found that testosterone levels improved with exercise programs, while cortisol reflected stress, which indicated frailty [34]. Immunoglobulin A (IgA) and lysozyme are both linked to immune function, with changes reflecting improvements in immune balance and frailty reduction [33,34].

## 4. Discussion

Frailty is characterized by a decrease in physical strength, mobility, and cognitive decline and presents a major issue in aging populations. Promoting healthy aging and maintaining functional independence are essential goals for older adults in enhancing their well-being and quality of life. Therefore, identifying effective exercise program interventions to prevent or delay frailty progression is important. This systematic review exhibited the significant effects of exercise interventions on reducing frailty and improving physical, cognitive, and emotional outcomes, as well as biomarker changes in older adults. In this study, the potential of multicomponent exercise programs to reverse or reduce frailty and improve overall well-being, together with the changes in biomarkers, is demonstrated through the analysis of results from multiple studies. All study settings, including community-dwelling, health centers, institutionalized, and hospitalized centers, were considered in order to target a diverse older population.

The primary outcome observed in the majority of studies was a reduction in physical frailty, as assessed by standardized frailty measures such as FFP, EFS, SPPB, and the FFP index. In their studies, Tarazona-Santabalbina et al. and Sadjapong et al. showed that frailty was either reversed or significantly reduced after participating in multicomponent exercise programs [28,31]. The programs combined endurance, strength, balance, and flexibility exercises, which were important for maintaining muscle mass, coordination, and cardiovascular health in frail older adults [26]. Reducing frailty is vital because it is related to negative health outcomes such as the increased risk of falls, hospitalization, disability, and mortality. Reversing or slowing the progression of frailty results in older adults retaining their functional capacity and enhancing their quality of life for prolonged periods [39]. An improvement in physical function was another critical primary outcome. This was assessed using HGS, TUG, BBS, and GST, which are widely recognized as predictors of mobility, independence, and fall risk in older adults. For instance, in the study by Sadjapong et al., frail participants experienced significant improvements in HGS and balance following a multicomponent exercise program [28]. Similarly, Furtado et al. reported enhanced balance and mobility, measured by improvements in TSBT and GST [34]. The observed improvements relate directly to the participants’ abilities in performing activities of daily living (ADLs), including walking, standing from a seated position, and maintaining balance during movement [40]. The significant improvement of the above physical outcomes suggests the importance of multicomponent exercise programs in enhancing functional independence and reducing fall risk, which are both necessary to prevent the adverse health trajectories frequently observed in frail older adults.

Several previous studies have demonstrated similar beneficial effects, supporting the effectiveness of the multicomponent exercise intervention across various populations. Multicomponent exercise programs combine aerobic, resistance, balance, and flexibility exercises, improving cardiovascular fitness, muscle strength, stability, and joint mobility. The variety of these exercises is essential for older adults, particularly those experiencing frailty. In contrast, single-component exercise usually emphasizes a specific area, such as aerobic or resistance training, indicating that it may not completely meet the overall criteria for older adults with frailty. In 2013, researchers conducted a large-scale study which presented that multicomponent exercise interventions are more effective than single-exercise practices in improving physical performance and preventing the development of frailty [7]. Other studies revealed that frail older adults who performed in a multicomponent exercise program demonstrated enhanced physical performance and reduced frailty and risk of falling [41,42]. The SPRINTT Trial, a major European study, demonstrated that multicomponent exercise interventions, including strength, balance, and endurance training, can effectively delay the onset of disability and frailty in older adults [43]. Along with previous systematic reviews, they similarly emphasized the effectiveness of multicomponent exercise interventions in managing frailty [40,44]. These reviews supported the concept that integrating different exercise types can markedly improve physical function and reduce frailty in older people. Their findings strongly coincide with our results, therefore supporting the use of planned or multicomponent exercise programs as a vital strategy for the prevention and management of frailty. Additionally, some research has shown that combining physical exercise with other interventions, such as nutritional supplementation or cognitive training, could effectively manage frailty and improve overall health and resilience in older adults [21,45]. While some studies in our analysis combined cognitive training with an exercise program, they did not explore the potential benefits of nutritional supplementation. The combined effect of the findings from previous studies promotes the fact that multicomponent exercise programs provide an effective intervention for reducing frailty in older adults. These programs enhance physical performance and cognitive and emotional well-being by targeting several aspects of physical and functional health, resulting in a significant reduction in frailty and an improved overall quality of life.

Another key primary outcome observed in several studies was the enhancement of cognitive and emotional well-being. Cognitive frailty, defined as the presence of physical and cognitive impairments, was particularly responsive to exercise, leading participants to improved mental capacity and reduced cognitive decline [46,47]. Tarazona-Santabalbina et al. and Zuyun Liu et al. highlighted significant enhancements in cognitive performance, as measured by tools such as the MMSE and 3MSE [31,32]. These findings suggest that multicomponent exercise can help slow or reverse cognitive decline in frail older adults, probably due to the improved cerebral blood flow and neurogenesis stimulated by physical activity. This cognitive improvement could be attributed to increased BDNF levels, which promote neuroplasticity and cognitive resilience [31]. Similar to the previous study, the findings indicate that physical activity intervention, particularly aerobic and strength training, improves cognitive function, mostly in memory and attention, along with showing a protective effect on cognitive health by increasing cerebral blood flow and stimulating neurogenesis [48]. Another previous study found that a multicomponent exercise program, including aerobic, resistance, and cognitive training, significantly improved attention, memory, and executive function in older adults with mild cognitive impairment [49]. The findings suggested that multicomponent exercise programs can positively influence brain health, making exercise a viable intervention for preventing or delaying cognitive decline in frail populations.

The outcomes from the studies usually point to an improvement in emotional well-being. Tarazona-Santabalbina et al. and Furtado et al. reported that depression symptoms were significantly decreased and life satisfaction was significantly increased after an exercise program [31,34]. The use of tools such as the GDS and SWLS showed that older adults participating in exercise experienced greater emotional stability, reduced loneliness, and improved social support. In 2001, the research literature suggested that regular practice in multicomponent exercise programs enhanced emotional heath through promoting social connection and self-confidence as well as minimizing isolation [50]. Another prior study revealed that multicomponent exercise improved mood and reduced anxiety levels [51]. Therefore, the findings imply that physical activity not only enhances physical and cognitive health but also supports emotional well-being and social links, which are key components of healthy aging and quality of life.

The physiological mechanisms that contributed to frailty reduction included improvements in muscle mass and strength, as well as enhanced cardiovascular fitness, all of which resulted in more effective physical performance and mobility. The secondary outcome emphasized biomarkers associated with frailty, providing a greater understanding of how exercise interventions improve several health functions in frail older adults. One of the most significant secondary outcomes was the decrease in pro-inflammatory biomarkers, which are closely correlation with the aging process and frailty. Frailty is often accompanied by a chronic low-grade inflammatory state known as inflammaging, which is characterized by elevated levels of markers like IL-6, TNF-α, and CRP. A variety of studies, including those by Zuyun Liu et al. and Sadjapong et al., found that multicomponent exercise programs significantly reduced IL-6 levels [28,32]. As we know, IL-6 is a key pro-inflammatory cytokine associated with age-related diseases and frailty [52,53]. Elevated IL-6 levels are associated with muscle weakness, impaired mobility, and cognitive decline [54]. The reduction in IL-6 following exercise interventions indicates that multicomponent exercise could impact the systemic inflammation that contributes to frailty. In studies by CaldoSilva et al. and L.F. Tan et al., TNF-α, another key inflammatory marker, was shown to decrease after an exercise program [35,37]. TNF-α is involved with frailty by promoting inflammation and muscle catabolism or sarcopenia [55,56]. Therefore, exercise interventions contribute to preventing muscle degradation and improving overall physical function by reducing TNF-α levels. CRP is a general marker of inflammation that is implicated in the pathogenesis of several age-related conditions [57]. Sadjapong et al. reported significant reductions in CRP levels in frail older adults after participating in a multicomponent exercise program [28]. Since a high level of CRP has been associated with an increased risk of cardiovascular disease and mortality in frail older adults, decreasing CRP levels is particularly important [58]. The lowering of CRP levels not only improves physical outcomes but also significantly impacts the underlying biological processes that lead to frailty. This confirms that regular physical activity could delay the progression of frailty and improve overall health by reducing systemic inflammation. Our findings demonstrated an association between biomarkers and frailty as compared with other systematic reviews, indicating a significant correlation between CRP and IL-6 inflammatory biomarkers and frailty in older adults, particularly seen in reviews that use Fried’s frailty phenotype [52]. Nonetheless, our study focused on exercise program interventions and did not directly assess biomarkers; understanding this biological relation enhances our discussion on how exercise programs might assist in reducing frailty by potentially decreasing inflammation. This may further explain how exercise programs reduce frailty, both functionally and biologically.

Furthermore, hormonal balance and oxidative stress markers were studied, both of which relate to the aging process and the development of frailty. Furtado et al. observed improvements in salivary testosterone levels in frail older women following a chair-based exercise program [34]. The decline of testosterone levels, a key hormone that maintains muscle mass, strength, and functional capacity, is a key cause of sarcopenia in aging populations [59]. However, this finding indicates that increasing testosterone levels is related to benefits in physical performance and emotional well-being [34]. Cortisol levels did not significantly change in the study [34]. Maintaining lower cortisol levels through exercise or physical activity might contribute to greater emotional stability, muscle strength, and cognitive improvement [60]. Moreover, protein carbonylation, MDA, 8-iso-PGF2α, and SOD were the biomarkers of oxidative stress studied by Tarazona-Santabalbina et al. and Yu Ye et al. [31,38]. These biomarkers present a vital understanding of the oxidative damage that occurs during the aging process, playing a role in frailty and age-associated diseases [61,62]. Exercise interventions were shown to reduce oxidative stress, as evidenced by decreases in MDA and 8-iso-PGF2α levels, as well as increases in antioxidant enzymes such as SOD [38]. It can be concluded that the reduction in oxidative damage assists in protecting cells and tissues, which promote a reverse of frailty. In addition, coagulation and hematological markers were elevated in two studies [31,36]. Tarazona-Santabalbina et al. reported that the levels of D-dimer significantly decreased in multicomponent exercise intervention, which related to a decreased risk of clotting and cardiovascular disease-associated frailty [31]. Previous evidence has explained that the D-dimer is linked to adverse cardiovascular outcomes and frailty, while physical activity or exercise decreases D-dimer levels [63,64]. The study by CaldoSilva et al. also presented that exercise programs improve immunological and red blood cell health, which are crucial for improved health and reduced frailty [36]. The findings suggest that multicomponent exercise programs exert broader effects on physiological systems, consisting of hormonal regulation, oxidative stress reduction, coagulation reduction, and hematological markers change, all of which are essential to prevent frailty.

This systematic review aimed to find a correlation between primary outcomes such as reductions in frailty, improved physical function, and enhanced cognitive and emotional well-being, as well as secondary outcomes of determining biomarker-associated frailty. The summarized results from several studies concluded that multicomponent exercise programs alter biomarkers associated with frailty through decreasing inflammatory and oxidative stress, positively impacting neuroplasticity, balancing hormonal markers, and others effects, resulting in a reduction in frailty, improvements in physical performance and cognitive function, and enhanced emotional well-being. The physiological mechanisms of biomarkers show how multicomponent exercise programs revers frailty and promote healthy aging and improved quality of life in frail older adults.

Recently, there has been an increase in research on aging, particularly exploring the impact of multicomponent exercise and biomarkers on frailty. Most systematic reviews emphasized solely the impact of multicomponent exercise or biomarkers on frailty; however, there are limited systematic reviews investigating the relationship between multicomponent exercise and biomarkers in the context of frailty. A systematic review by CaldoSilva and colleagues reported results that align with our findings. Their study, conducted between 2001 and 2020, primarily focused on physical outcomes and specific inflammatory markers, such as IL-6, TNF-α, and CRP, in response to multicomponent exercise [65]. Meanwhile, our findings, including updated data between 2014 and 2024, encompassed a broader range of clinical outcomes, including frailty, physical performance, cognitive function, emotional well-being, and social factors. Additionally, our study provided some different biomarkers and a more in-depth analysis, including inflammatory markers, oxidative stress biomarkers, and hormonal and immune biomarkers (Table 2), offering a more complete understanding of the physiological impacts of exercise. Therefore, this study bridges the gap between the clinical symptoms of frailty and the underlying biological mechanisms.

## 5. Limitations and Future Directions

Most of the studies included focused on health centers, care homes, and community-dwelling older adults, with limited investigations on hospitalized frail persons who may respond differently to exercise programs. While the review provides strong evidence for the benefits of exercise interventions, there are several limitations. Furthermore, a variety of exercise program intensity, duration, and adherence rates among participants may influence outcomes, suggesting a requirement for standardized procedures. Future studies should explore the long-term effectiveness of exercise-induced improvements in frailty, as well as the particular mechanisms by which exercise impacts cognitive function and inflammatory pathways. It would also be important to study the potential significance of personalized exercise routines suited to individual frailty profiles, ensuring that interventions are both safe and effective across various groups.

## 6. Conclusions

The findings of this review emphasize the critical role of multicomponent exercise programs in reducing frailty, improving physical function, and enhancing cognitive and emotional well-being, as well as focusing on biomarker-associated frailty change in older adults. Exercise program interventions provide a systemic approach to managing the complex syndrome of frailty by focusing on both physical and cognitive variables. These results highlight the importance of using multicomponent exercise in care plans for pre-frail and frail older adults, not only to improve their physical health but also to enhance their mental and emotional well-being, thereby contributing to healthy aging. The findings provide practical applications for clinical practice by supporting healthcare professionals in developing and suggesting exercise programs which are suitable for older adults. Therefore, exercise programs enable clinicians to effectively manage frailty, slow its progression, and enhance independence among older adults.

## Figures and Tables

**Table 1:** Review characteristics of individual studies.

Author/ Country/ Year	Mean of Age (Number of Participants)	Study Setting	Frailty Status	Activities/Intensity		Assessment Tools		Biomarkers	Outcomes
				Exercise Program Intervention	Control Group	Frailty	Others		
F.J. Tarazona-Santabalbina, et.al, Spain, 2016 [31]	I: 79.5 ± 3.9 (51) C: 80.3 ± 3.7 (49)	Primary health center	Frail	Multicomponent exercise program	No training and attending the regular primary care program	FFP, EFS	SPPB, PPT, Barthel, Lawton, Tinetti, TUG, Tinetti gait test, Tinetti balance index, FAC, PAE, MMSE, Duke social support, Yesavage, EQ-5D	Serum D-Dimer, Protein carbonylation, BDNF	Primary outcome Reversing of frailty Improvement of functional measurements Improvement of cognition, emotional, and social state of individuals Secondary outcome Significantly improved biomarkers of frailty
Zuyun Liu, et.al, USA, 2018 [32]	I: 78.6 ± 5.2 (647) C: 79.1 ± 5.3 (656)	Multicenter (8 US field centers)	Cognitive frailty	Multicomponent exercise	Health education program for sedentary older persons	SOF index	SPPB, 3MSE	Plasma IL-6	Primary outcome Improvement in cognitive frailty Secondary outcome IL-6 level did not change
Sadjapong, et.al, Thailand, 2020 [28]	I: 76.68 ± 1.14 (32) C: 78.87 ± 1.32 (32)	Community-dwelling	Frail	Multicomponent exercise program	Usual care	FFP	MMST10, HGT, BBS, TUG, VO_2_ max, HRQOL	Serum IL-6, CRP	Primary outcome Improved physical performance Reduced frailty Secondary outcome Reduced inflammatory biomarkers
Eustáquio, et.al, Portugal, 2020 [33]	I: 80 ± 8.19 (20) C: 80 ± 10.01 (19) (Women)	Care home	Pre-frail and frail with mild cognitive impairment	Functional circuit training protocol	No exercise (usual lifestyle)	FFP	The Senior Fitness Test battery, MMSE, MNA	Saliva sIg-A, sLys, IL-1β, IL-6 Serum TNF-α, IFN-γ, IL-10	Primary outcome Increased physical fitness and promoted independence in cognitively frail older women Secondary outcome Slight reduction in pro-inflammatory responses Balance of pro- and anti-inflammatory responses
Furtado, et.al, Portugal, 2021 [34]	I: 81.1 ± 7.5 (17) C: 83.3 ± 8.2 (15) (Women)	Social and health care	Pre-frail	Combined chair-based exercises	Non-exercising control group	HGT (FFP)	MNA, MMSE, SWLS, PSS, HFS, HGT, GST, TSBT	Salivary Testosterone, Cortisol, Ig-A, Lysozyme	Primary outcome Improved functional fitness and subjective well-being Secondary outcome Improved salivary testosterone levels
CaldoSilva, et.al, Portugal, 2021 [35]	I: 86.7 ± 4 (7) C: 83.1 ± 5.4 (13)	Public and private residential home care	Pre-frail and frail	Multicomponent exercise program Phase 1: Elastic band exercise Phase 2: Washout phase Duration: 8 weeks Phase 3: Multicomponent exercise	No regular exercise/no supplementation	FFP index	IPAG-SV, MNA, 5TSS, CCI, MMSE	Plasma/serum IL-10, TNF-α, MPO, Albumin	Primary outcome Improved cognitive profile Secondary outcomeInduced slight changes in pro-inflammatory marker TNF-α
CaldoSilva, et.al, Portugal, 2023 [36]	I: 86.7 ± 4 (7) C: 83.1 ± 5.4 (13)	Public and private residential home care	Pre-frail and frail	Multicomponent exercise program Phase 1: Elastic band exercise Phase 2: Washout phase Duration: 8 weeks Phase 3: Multicomponent exercise	No regular exercise/no supplementation	FFP index	IPAG-SV, MNA, 5TSS, CCI, MMSE	Blood Erythrogram, Leukogram, Platelet	Primary outcome Frailty decreased over time Secondary outcomePotential pro-immune effect and maintenance of MCH and MCHC levels
L.F. Tan, et.al, Singapore, 2023 [37]	I: 73.39 ± 5.20 (80) C: 71.69 ± 4.99 (187)	Primary care clinic	Pre-frail	Multicomponent exercise program	General health education advice	Frail scale	HGS, GST, SPPB, 5TSS, RAPA, MoCA, GDS, EQ-VAS, MNA-SF	Plasma TNF-α, IL-6, GDF-15, IL-10	Primary outcome Significantly improved frail total score Secondary outcomeTNF-α level was significantly reduced
Yu Ye, et.al, China, 2024 [38]	I: 67.68 ± 5.19 (51) C: 65.35 ± 5.15 (51)	Community-dwelling	Cognitive frailty	Exercise training (Baduanjin)	No specific exercise intervention	EFS (Chinese version)	MoCA	Serum MDA, SOD, 8-iso-PGF2α, NO, T-AOC. LPO, IL-2, IL-4, IL-6, IL-8, IL-10, TNF-α, IFN-γ	Primary outcome Improved cognition frailty Secondary outcome Reduced circulating pro-oxidative and increased anti-oxidative Impact on inflammatory cytokines

**Table 2:** The characteristics of exercise program interventions.

Author/Country/Year	Exercise Program	Components	Duration
F.J. Tarazona-Santabalbina, et al., Spain, 2016 [31]	Multicomponent exercise program	Warm-up Proprioception and balance exercises (postural sway and dynamic balance, coordination, and flexibility of the lumbo-pelvic area) Aerobic training (walking around a circuit and climbing stairs) Strength training (resistance bands and isometric, concentric, and eccentric exercises with the arms, hands, and legs.) Stretching exercises, including the arms, legs, and neck	65 min/session, 5 days/week for 24 weeks
Zuyun Liu, et al., USA, 2018 [32]	Multicomponent exercise	Walking daily at moderate intensity Primarily lower extremity strength training by means of body weight and ankle weights (2 sets of 10 repetitions) Large muscle group flexibility exercises Balance training	3–4 times/week for 24 months Two center-based visits a week Home-based activity, three to four times a week
Sadjapong, et al., Thailand, 2020 [28]	Multicomponent exercise program	Chair aerobic training Resistance training with a Theraband Balance training From moderate to high	60 min, 3 days/week for 24 weeks
Eustáquio, et al., Portugal, 2020 [33]	Chair multimodal exercise (Functional circuit training protocol)	Each session was conducted using a functional circuit training protocol. During the first 12 weeks, the body weight exercises were executed in the seat and reach positions. During the last 12 weeks, the exercise intensity increase was induced by the inclusion of more difficult and complex exercises and challenge sequences, increasing the walking time, and placing obstacles along the walking route (cones, floor markers, arcs) to work handedness, changes in direction, static/dynamic balance, and coordination.	2–3 sessions/week for 28 weeks
Furtado, et al., Portugal, 2021 [34]	Combined Chair-Based Exercises	Each session was divided into five parts: Warm-up and body mobilization Low/upper body elastic-band exercises Static and dynamic balance exercises Sequential exercises improving gait speed Stretching exercises as a “cool down” strategy	2–3 times/week for 14 weeks
Adriaana CaldoSilva, Portugal, et al., 2021 and 2023 [35,36]	Multicomponent exercise program	Phase 1: Elastic band exercise Front squat Chair unilateral hip flexion Chair bench over row (with flexion) Chest press (stand and/or chair) Standing (or chair) reverse fly Shoulder press/twist arm position Chair (or stand) frontal total raiser Biceps arm curl (stand and/or chair) Chair overhead triceps extension Phase 2: Washout phase (exercise detraining) Phase 3: Multicomponent exercise Front squat Chair unilateral hip flexion Chair bench over row (with flexion) Chest press (stand and/or chair) Standing (or chair) reverse fly Shoulder press/twist arm front position Chair (or stand) frontal total raiser Biceps arm curl (stand and/or chair) Chair overhead triceps extension Circuit training Walking around room Balance/agility exercise	40-week multifactorial intervention Phase 1: twice a week with an interval of 36 h for 16 weeks Phase 2: 8 weeks Phase 3: twice a week, on alternate days, 32 sessions for 16 weeks
L.F. Tan, et al., Singapore, 2023 [37]	Multicomponent exercise program	Aerobic training Resistance training Dual task Balance training	60 min twice a week, for 12 months
Yu Ye, et al., China, 2024 [38]	Exercise training	Baduanjin (10 postures including preparation and ending posture)	60 min 3 days/week for 24 weeks

**Table 3:** Categories of biomarker associated with frailty, as seen in exercise program interventions.

Categories	Biomarker Associated Frailty
Inflammatory biomarker	IL-6, IL-1β, IL-2, IL-4, IL-8, IL-10, CRP, IFN-γ, TNF-α, MPO, GDF-15
Oxidative stress biomarker	Protein carbonylation, MDA, 8-iso-PGF2α, SOD, T-AOC, NO
Coagulation biomarker	D-dimer
Neurotrophic factors	Brain-derived neurotrophic factor (BDNF)
Hormonal biomarker	Testosterone, Cortisol
Immune system biomarker	IgA, Lysozymes
Metabolic and protein biomarker	Albumin
Hematological biomarker	Erythrogram, Leukogram, Platelet

## Abbreviations

3MSE = modified mini-mental state examination, 8-iso-PGF2α = 8-iso-prostaglandin F2 alpha, BBS = Berg balance scale, BDNF = brain-derived neurotrophic factor, C = control, CRP = C-reactive protein, EFS = Edmonton frailty scale, EQ-5D = EuroQol quality-of-life scale, FAC = functional ambulation categories, FFP = Fried’s frailty phenotype, HRQOL = health-related quality of life, HFS = happiness face scale, HGT = hand grip strength test, GDF-15 = growth/differentiation factor 15, GDS = geriatric depression scale, GST = gait speed test, I = intervention, IFN-γ = interferon-gamma, Ig-A = immunoglobulin-A, IL-1β = interleukin-1 beta, IL-2 = interleukin-2, IL-4 = interleukin-4, IL-6 = interleukin-6, IL-8 = interleukin-8, IL-10 = interleukin-10, IPAG-SV = international physical activity questionnaire, short version, LPO= lipid peroxidation, Lys = lysozyme, MDA = malondialdehyde, MNA = mini nutritional assessment, MMSE = mini-mental state examination, MMST10 = mini-mental state examination—Thai version, MPO = myeloperoxidase activity, NO = nitric oxide, RAPA = rapid physical assessment, SOD = superoxide dismutase, SOF = study of osteoporotic fractures, SPPB = short physical performance battery, SWLS = satisfaction with life scale, PPT = physical performance test, PSS = perceived stress scale, T-AOC = total antioxidant capacity, TNF-α = tumor necrosis factor alpha, TSBT = tandem stance balance test, TUG = timed-up-and-go.
